# Associations between Source-Specific Fine Particulate Matter and Emergency Department Visits for Respiratory Disease in Four U.S. Cities

**DOI:** 10.1289/EHP271

**Published:** 2016-06-17

**Authors:** Jenna R. Krall, James A. Mulholland, Armistead G. Russell, Sivaraman Balachandran, Andrea Winquist, Paige E. Tolbert, Lance A. Waller, Stefanie Ebelt Sarnat

**Affiliations:** 1 Department of Biostatistics and Bioinformatics, Emory University, Atlanta, Georgia, USA; 2 School of Civil and Environmental Engineering, Georgia Institute of Technology, Atlanta, Georgia, USA; 3 Department of Biomedical, Chemical & Environmental Engineering, University of Cincinnati, Cincinnati, Ohio, USA; 4 Department of Environmental Health, Emory University, Atlanta, Georgia, USA

## Abstract

**Background:**

Short-term exposure to ambient fine particulate matter (PM2.5) concentrations has been associated with increased mortality and morbidity. Determining which sources of PM2.5 are most toxic can help guide targeted reduction of PM2.5. However, conducting multicity epidemiologic studies of sources is difficult because source-specific PM2.5 is not directly measured, and source chemical compositions can vary between cities.

**Objectives:**

We determined how the chemical composition of primary ambient PM2.5 sources varies across cities. We estimated associations between source-specific PM2.5 and respiratory disease emergency department (ED) visits and examined between-city heterogeneity in estimated associations.

**Methods:**

We used source apportionment to estimate daily concentrations of primary source-specific PM2.5 for four U.S. cities. For sources with similar chemical compositions between cities, we applied Poisson time-series regression models to estimate associations between source-specific PM2.5 and respiratory disease ED visits.

**Results:**

We found that PM2.5 from biomass burning, diesel vehicle, gasoline vehicle, and dust sources was similar in chemical composition between cities, but PM2.5 from coal combustion and metal sources varied across cities. We found some evidence of positive associations of respiratory disease ED visits with biomass burning PM2.5; associations with diesel and gasoline PM2.5 were frequently imprecise or consistent with the null. We found little evidence of associations with dust PM2.5.

**Conclusions:**

We introduced an approach for comparing the chemical compositions of PM2.5 sources across cities and conducted one of the first multicity studies of source-specific PM2.5 and ED visits. Across four U.S. cities, among the primary PM2.5 sources assessed, biomass burning PM2.5 was most strongly associated with respiratory health.

**Citation:**

Krall JR, Mulholland JA, Russell AG, Balachandran S, Winquist A, Tolbert PE, Waller LA, Sarnat SE. 2017. Associations between source-specific fine particulate matter and emergency department visits for respiratory disease in four U.S. cities. Environ Health Perspect 125:97–103; http://dx.doi.org/10.1289/EHP271

## Introduction

Many epidemiologic studies have reported positive associations between short-term exposure to ambient fine particulate matter (PM) air pollution, PM < 2.5 μm in aerodynamic diameter (PM_2.5_), and increased mortality and morbidity (Dominici et al. 2006; Samoli et al. 2013; Stafoggia et al. 2013). PM_2.5_, constituents of which include metal oxides, sulfate, organic carbon (OC), and elemental carbon (EC) (Bell et al. 2007), varies geographically in chemical composition depending on its natural and/or anthropogenic generating sources (Hackstadt and Peng 2014; Hopke et al. 2006). Individual PM_2.5_ chemical constituents vary in their associations with adverse health outcomes (Krall et al. 2013; Ostro et al. 2008; Sarnat et al. 2015). Because PM_2.5_ sources emit mixtures of chemical constituents, source-specific PM_2.5_ also varies in its associations with adverse health outcomes (Ito et al. 2006; Mar et al. 2006; Sarnat et al. 2008). Estimated associations between source-specific PM_2.5_ and health have varied among previous studies, which have primarily used data from one city or from a few communities (Bell et al. 2014; Mar et al. 2006; Sarnat et al. 2008). Multicity studies provide the means to fully compare estimated associations of source-specific PM_2.5_ across cities. Understanding which PM_2.5_ sources are the most toxic could help inform targeted reduction and possibly regulation of ambient PM_2.5_, which at the present time is regulated by total mass concentration via the U.S. National Ambient Air Quality Standards (NAAQS).

Conducting epidemiologic studies of PM_2.5_ sources is challenging because source-specific ambient PM_2.5_ cannot be directly measured and must be estimated using methods such as source apportionment models. Standard source apportionment models estimate source-specific PM_2.5_ separately for each ambient monitor using PM_2.5_ constituent concentrations. In multicity studies, PM_2.5_ sources estimated separately at each ambient monitor must be matched between monitors, which is difficult because PM_2.5_ sources can vary between cities in both chemical composition and concentration (Ito et al. 2004; Sarnat et al. 2008). For cities located far apart from each other, the chemical composition of some PM_2.5_ sources may vary between cities because of local differences in industry, types of vehicles, or other factors.

Previously observed city-to-city heterogeneity in PM–health associations (Samet et al. 2000; Franklin et al. 2007) may be driven by differences in population or exposure characteristics, such as susceptibility or air conditioning use, respectively, or by differences between cities in the chemical composition of source-specific PM_2.5_. We can eliminate some of this between-city variation by only comparing estimated health effect associations of sources whose chemical compositions do not vary substantially between cities. By restricting our analysis to sources with similar chemical composition across cities, we can better compare estimated health effect associations of the same exposures, that is to say, source-specific PM_2.5_, across cities.

Although most U.S. studies of source-specific PM_2.5_ have used data from only one or two ambient monitors (Hopke et al. 2006; Sarnat et al. 2008), a few multicommunity epidemiologic studies of source-specific PM_2.5_ have been conducted. Bell et al. (2014) estimated source-specific PM_2.5_ using data from five ambient monitors in Massachusetts and Connecticut, although the monitors were located in four contiguous counties and likely measured similar sources. Ito et al. (2013) estimated source-specific PM_2.5_ across 64 U.S. cities but did not quantify how similar the sources were between cities. Although these multicity studies estimated associations between source-specific PM_2.5_ and health, a more comprehensive evaluation of how the chemical composition of PM_2.5_ sources varies across cities is still needed.

We estimated associations between short-term exposure to source-specific PM_2.5_ and respiratory disease emergency department (ED) visits for four U.S. cities: Atlanta, Georgia; Birmingham, Alabama; St. Louis, Missouri; and Dallas, Texas. These cities, which are located in the southern and midwestern United States, likely have some PM_2.5_ sources that are similar in chemical composition across cities, but others may differ because of the presence of different industries, varying meteorology, or other factors. We focused on primary PM_2.5_ sources, such as traffic and coal combustion, which emit PM_2.5_ directly. Separately for each city, we estimated source-specific PM_2.5_ and then identified those sources with similar chemical compositions across cities. For similar sources, we estimated associations between source-specific PM_2.5_ and respiratory disease ED visits. In this paper, we report how source apportionment results can be compared between cities in epidemiologic studies of air pollution, and we present the first multicity U.S. study of the associations between primary source-specific PM_2.5_ and respiratory disease ED visits.

## Methods

### Data

We obtained electronic billing data for respiratory disease ED visits for all ages at acute care hospitals in the 20-county Atlanta metropolitan area, the 7-county Birmingham metropolitan area, the 8 Missouri and 8 Illinois counties in the St. Louis metropolitan area, and the 12-county Dallas metropolitan area. Previous studies described the data collection for Atlanta (Sarnat et al. 2010) and St. Louis (Sarnat et al. 2015). Using diagnosis codes from the *International Classification of Diseases, 9th Revision* (ICD-9), we considered subcategories of respiratory diseases including pneumonia (ICD-9 codes 480–486), chronic obstructive pulmonary disease (COPD) (491, 492, 496), upper respiratory infection (URI) (460–465, 466.0, 477), and asthma and/or wheeze (493, 786.07). We created a combined category of daily respiratory disease ED visits by adding the number of daily ED visits for these subcategories and including additional ICD-9 codes for bronchiolitis (466.1, 466.11, 466.19). We used ED visit data in accordance with our data use agreements with the Georgia Hospital Association, the Missouri Hospital Association, the Dallas–Fort Worth Hospital Council Foundation, and selected individual hospitals. The Emory University Institutional Review Board approved this study and granted an exemption from informed consent requirements, given the minimal risk nature of the study and the infeasibility of obtaining informed consent from individual patients for > 1.8 million billing records.

We obtained concentrations for PM_2.5_ mass and PM_2.5_ constituents from one urban, ambient monitor located in each city for the following time periods: Jefferson Street in Atlanta from 1999–2009, North Birmingham in Birmingham from 2004–2010, Blair Street in St. Louis from 2001–2007, and Hinton Street in Dallas from 2006–2009.

Daily pollution data were available in Atlanta; however, data were only available approximately every third day in the remaining three cities. To ensure estimated sources more closely resembled known PM_2.5_ sources, our source apportionment models incorporated additional data including concentrations of gaseous pollutants and, when available, the Community Multiscale Air Quality with Tracers (CMAQ-TR) model (Baek 2009). We obtained meteorological data for each city, including temperature and relative humidity, from the National Climatic Data Center.

### Source Apportionment

Source apportionment models generally assume that observed PM_2.5_ constituent concentrations *X* are formed as a linear combination of *source profiles Λ*, the chemical composition of each source, and daily *concentrations of source-specific PM_2.5_ F*, plus some independent error ε; that is, *X = ΛF + ε*. We used an ensemble approach to estimate city-specific ensemble-based source profiles (EBSPs). The EBSPs are then used in chemical mass balance with gas constraints (CMB-GC) to estimate concentrations of source-specific PM_2.5_, a process which is described in detail elsewhere (Balachandran et al. 2012; Lee et al. 2009).

To estimate *source profiles* for each city, the EBSP approach uses a weighted average of several source apportionment models. Because of the variations in available information across cities, we used a different set of source apportionment models for each city, including CMB with molecular markers (Atlanta and St. Louis), CMB-GC (Marmur et al. 2005) (all cities), the CMAQ-TR model (Atlanta, Birmingham, and St. Louis), positive matrix factorization (PMF) (Paatero and Tapper 1994) (all cities), and PMF using molecular markers (St. Louis). These source apportionment methods have been used in other studies of source-specific PM_2.5_ and are described elsewhere (Maier et al. 2013; Sarnat et al. 2008). By using multiple source apportionment methods in each city, we were able to leverage the advantages of each method. To account for differences in source-specific PM_2.5_ between summer and winter months, EBSPs were estimated separately for warm and cold seasons using data from July and January, respectively. Two months were used because these were the only months for which results were available for CMAQ and CMB with molecular markers.

*Concentrations of source-specific PM_2.5_* were estimated separately for each city using CMB-GC, which uses gaseous pollutants to improve estimates of source-specific PM_2.5_ (Marmur et al. 2005). The winter EBSPs were used to estimate concentrations of source-specific PM_2.5_ for November through March, and the summer EBSPs were used to estimate concentrations for the remaining months. Because the same approach (CMB-GC) was used to estimate source concentrations for each city, sources with similar EBSPs were compared between cities despite incorporating different source apportionment methods. Although secondary PM_2.5_ sources were not the focus of this study, source profiles for secondary sources were also included in the CMB-GC.

To assess similarity among the chemical compositions of source-specific PM_2.5_ across cities, we compared the proportions of each PM_2.5_ constituent in each source using normalized root mean squared differences (nRMSDs) of the EBSPs, which were normalized by the average range (maximum–minimum) within EBSPs for each source (Marzo 2014). We also used correlations to indicate whether PM_2.5_ constituents in each estimated source were linearly associated. The correlations and nRMSDs were computed by comparing EBSPs for a particular source between two cities, separately for winter and summer EBSPs, and summarizing across pairwise comparisons between cities for each season using the average, minimum, and maximum values. To assess similarity between EBSPs for each source, we used a 10% cutoff in the maximum nRMSD across pairwise comparisons.

### Associations with ED Visits

To estimate associations between short-term exposure to source-specific PM_2.5_ and respiratory disease ED visits, we applied overdispersed Poisson time-series regression models to data from each city controlling for potential confounders as in previous studies of PM_2.5_ and cardiorespiratory ED visits (Winquist et al. 2015). Specifically, we included indicator variables for holidays, day of the week, season, and the hospitals reporting data for each day. We controlled for meteorology using separate cubic polynomials for same-day (lag 0) maximum temperature, the mean of previous-day and 2 days before (lags 1–2) minimum temperature, and the mean of lags 0–2 dewpoint temperature. We controlled for long-term trends in ED visits using cubic splines of time with one degree of freedom per month. Last, we incorporated pairwise interaction terms between season and each of the following: maximum temperature, weekdays, and federal holidays. We estimated associations separately for each source for single-day exposures at lags 0, 1, 2, and 3. Because we did not have daily source-specific PM_2.5_ concentrations for Birmingham, St. Louis, and Dallas, we could not estimate exposures across multiple days. We scaled the resulting relative risks by the median of the city-specific interquartile ranges (IQR) corresponding to each source. We only estimated associations between source-specific PM_2.5_ and ED visits for those sources that had similar chemical compositions across cities based on the nRMSD. We compared estimated health effect associations across cities using chi-squared tests of heterogeneity (Kleinbaum et al. 1982; Rothman et al. 2008).

The estimated chemical composition of source-specific PM_2.5_ from source apportionment models may not correspond well to the true source chemical composition in each city. We explored an alternative approach by estimating health effect associations corresponding to individual “tracer” PM_2.5_ chemical constituents known to be emitted from various PM_2.5_ sources. Inconsistencies between estimated associations of source-specific PM_2.5_ and estimated associations of tracer PM_2.5_ constituents may indicate that estimated source-specific PM_2.5_ may not correspond well to known PM_2.5_ sources.

### Sensitivity Analysis

As a sensitivity analysis, we estimated associations separately for subcategories of respiratory diseases including pneumonia, COPD, URI, and asthma/wheeze. To determine whether our results were sensitive to the confounders included in our health effects regression models, we compared our results with those from models without product terms, without dewpoint temperature, without lag 1–2 minimum temperature, without season, without holidays, and without holidays and weekdays. To investigate possible exposure misclassification, we compared our analysis of ED visits for patients residing in all counties of the surrounding metropolitan area with analyses using only ED visits from patients residing in the county or counties closest to each city center, which contained the ambient monitoring site (DeKalb and Fulton Counties, Atlanta; Jefferson County, Birmingham; St. Louis County and St. Louis City, St. Louis; Dallas County, Dallas).

The EBSPs were derived based on the source apportionment results that could be obtained for each city. For example, some source apportionment models, such as CMB with molecular markers, require more data than we had readily available for some cities. To determine whether our results were sensitive to the varying combinations of source apportionment methods across cities, we also estimated source profiles using a standard CMB approach in each city.

## Results

### Source Apportionment

Across four U.S. cities, we identified six primary PM_2.5_ sources including biomass burning, diesel vehicles, gasoline vehicles, dust, coal combustion, and metals, although each source was not identified in all cities. We did not identify a coal combustion source in St. Louis, nor did we identify a metals source in Atlanta and Dallas, although the remaining sources were present in all four cities. The metals source is a composite source representing industrial facilities such as steel processing (Lee et al. 2006). The estimated city- and season-specific EBSPs, which are unitless but can be interpreted as the amount (in micrograms/cubic meter) of each constituent per microgram/cubic meter of source-specific PM_2.5_, are displayed in Figures S1 and S2. We summarized differences in EBSPs using *N* pairwise comparisons between cities for each season, which yielded *N* correlations and *N* nRMSDs for each source (Table 1). For the EBSPs corresponding to biomass burning, diesel vehicles, gasoline vehicles, and dust, the maximum nRMSD across pairwise comparisons was < 10% and their correlations were also close to 1, suggesting strong similarity in these sources across cities. The EBSPs for coal combustion and metals varied between cities, with the maximum nRMSD > 10% and smaller correlations; therefore, we did not compare their estimated associations with ED visits across cities (Table 1; see also Figures S1 and S2).

**Table 1:** A comparison of ensemble-based source profiles for warm and cold seasons for Atlanta, Georgia; Birmingham, Alabama; St. Louis, Missouri; and Dallas, Texas.

Source of PM_2.5_	Number of cities^*a*^	Correlation^*b*^	nRMSD (%)^*c*^	Pairwise comparisons^*d*^
Biomass burning	4	0.99 (0.97, 1.00)	4.20 (2.04, 6.35)	12
Diesel vehicles	4	1.00 (1.00, 1.00)	2.30 (1.44, 3.66)	12
Gasoline vehicles	4	1.00 (1.00, 1.00)	2.10 (0.93, 3.54)	12
Dust	4	1.00 (0.99, 1.00)	2.52 (1.20, 4.26)	12
Coal combustion	3	0.69 (0.48, 0.98)	23.80 (11.45, 30.65)	6
Metals	2	0.67 (0.59, 0.74)	38.77 (37.46, 40.08)	2
Notes: nRMSD, normalized root mean squared difference. ^***a***^Number of cities where each source was identified. ^***b***^Average (minimum, maximum) correlation between EBSPs across cities for each season. ^***c***^Average (minimum, maximum) percent nRMSD comparing EBSPs across cities for each season. ^***d***^Number of pairwise comparisons made for EBSPs between cities for each season.				

For each city, we estimated concentrations of source-specific PM_2.5_ for 3,624 days in Atlanta, 808 days in Birmingham, 728 days in St. Louis, and 332 days in Dallas. Table 2 shows the average concentrations and standard deviations (micrograms/cubic meter) of source-specific PM_2.5_ for each city. For primary PM_2.5_ sources, we found that the greatest concentrations corresponded to biomass burning. Correlations between concentrations of source-specific PM_2.5_ and PM_2.5_ mass were generally small to moderate (see Table S1).

**Table 2:** Average (standard deviation) concentration and median of city-specific interquartile ranges in micrograms/cubic meter for PM_2.5_ mass and primary source-specific PM_2.5_ for four U.S. cities.*^a^*

Pollutant	Atlanta, GA	Birmingham, AL	St. Louis, MO	Dallas, TX	IQR
PM_2.5_ mass	15.55 (7.82)	17.00 (9.25)	13.56 (7.07)	10.71 (4.62)	9.16
Biomass burning	1.60 (1.17)	1.05 (1.04)	1.31 (0.95)	1.36 (0.95)	0.95
Diesel vehicles	1.19 (1.16)	1.02 (1.32)	0.72 (0.80)	0.30 (0.52)	1.11
Gasoline vehicles	1.01 (0.94)	0.70 (0.75)	1.11 (0.61)	0.48 (0.38)	0.72
Dust	0.43 (0.44)	0.60 (0.72)	0.46 (0.69)	0.65 (1.08)	0.33
Coal combustion	0.13 (0.12)	0.23 (0.30)	—	0.01 (0.02)	0.13
Metals	—	0.64 (0.57)	0.23 (0.24)	—	0.43
Notes: IQR, interquartile range; PM_2.5_, fine particulate matter ^***a***^Available days of source-specific PM_2.5_: 3,624 days for Atlanta, Georgia; 808 days for Birmingham, Alabama; 728 days for St. Louis, Missouri; and 332 days for Dallas, Texas.					

### Associations with ED Visits

The average number of daily ED visits for combined respiratory diseases was 361 (standard deviation = 129) for Atlanta, 59 (27) for Birmingham, 281 (81) for St. Louis, and 455 (159) for Dallas (see Table S2). In each city, the majority of daily respiratory disease ED visits were for URI.

**Figure 1:**
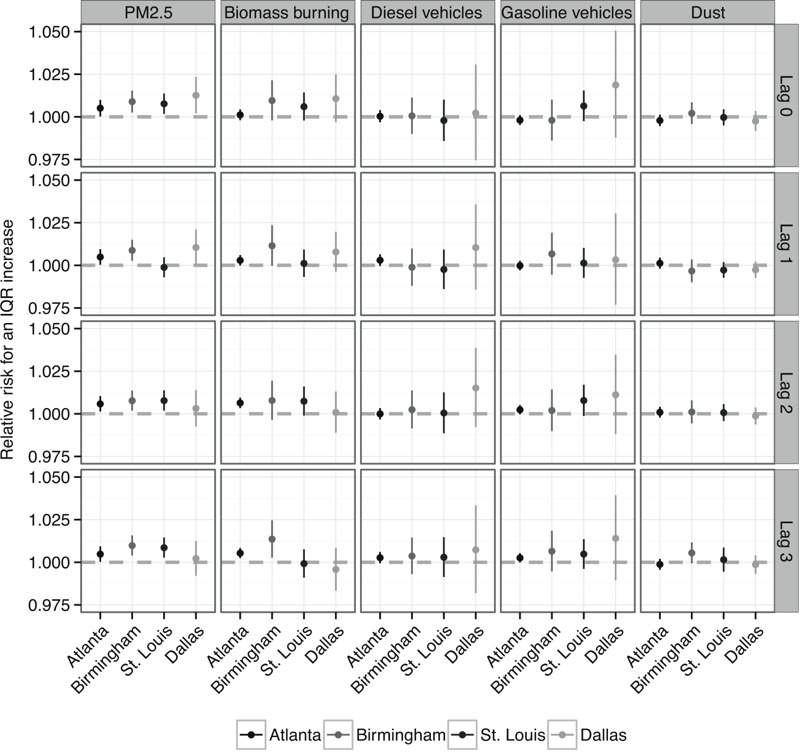
Estimated relative risks of respiratory disease emergency department (ED) visits for interquartile range (IQR) increases in fine particulate matter (PM_2.5_) mass and source-specific PM_2.5_ using single-day exposure lags 0 to 3 for Atlanta, Georgia; Birmingham, Alabama; St. Louis, Missouri; and Dallas, Texas.

Figure 1 shows the estimated relative risks and 95% confidence intervals (CIs) for an interquartile range (IQR) increase in PM_2.5_ mass and PM_2.5_ from biomass burning, diesel vehicles, gasoline vehicles, and dust for single-day lags 0 to 3. We did not compare associations across cities for PM_2.5_ from coal combustion or metals because their EBSPs varied substantially between cities (Table 1). For PM_2.5_ mass, associations with respiratory disease ED visits were frequently positive and statistically significant across cities, although the lag of greatest association varied between cities. For lag 2, the relative risk of respiratory disease ED visits associated with an IQR increase in PM_2.5_ mass was 1.006 (95% CI: 1.001, 1.010) for Atlanta, 1.008 (95% CI: 1.002, 1.014) for Birmingham, 1.008 (95% CI: 1.002, 1.014) for St. Louis, and 1.003 (95% CI: 0.993, 1.014) for Dallas. Associations for PM_2.5_ from biomass burning were positive and were frequently greater in magnitude than for other sources. The relative risk associated with an IQR increase in lag 2 PM_2.5_ from biomass burning was 1.006 (95% CI: 1.003, 1.010) for Atlanta, 1.008 (95% CI: 0.996, 1.019) for Birmingham, 1.007 (95% CI: 0.999, 1.016) for St. Louis, and 1.001 (95% CI: 0.989, 1.013) for Dallas.

For PM_2.5_ from diesel vehicles and gasoline vehicles, estimated associations were inconsistent across cities and lags with many near-null associations or associations with large standard errors. Across lags, the estimated associations in St. Louis were more positive for gasoline vehicles than for diesel vehicles. Associations with diesel and gasoline vehicles in Dallas had larger confidence intervals than other sources, which may be explained by the relatively low temporal variability of PM_2.5_ from these sources. Across cities and exposure lags, we did not find evidence that PM_2.5_ from dust was associated with respiratory disease ED visits. Using chi-squared tests of heterogeneity, we did not find evidence that estimated associations differed across cities for any PM_2.5_ source at any lag.

We selected tracer constituents to correspond to our identified sources based on Sarnat et al. (2008), including potassium for PM_2.5_ from biomass burning, EC for PM_2.5_ from diesel vehicles, zinc for PM_2.5_ from gasoline vehicles, and silicon for PM_2.5_ from dust. We also examined OC, which is emitted by biomass burning, diesel vehicles, and gasoline vehicles, but is not associated with dust PM_2.5_. Although none of these constituents is generated solely by the specified source categories, they can be used to help interpret the source-specific results. Tables S3–S6 summarize the data for PM_2.5_ constituent tracers, including correlations between source-specific PM_2.5_ and tracer constituents (Table S6).

**Figure 2:**
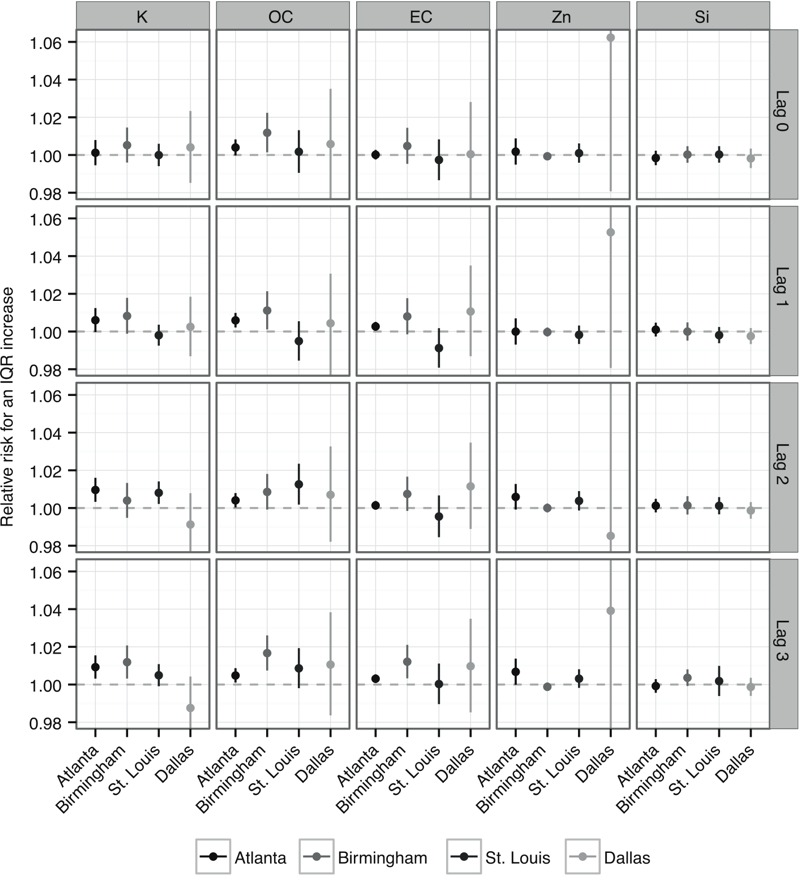
Estimated relative risks of respiratory disease emergency department (ED) visits for interquartile range increases in selected tracer fine particulate matter (PM_2.5_) constituents using single-day exposure lags 0 to 3 for Atlanta, Georgia; Birmingham, Alabama; St. Louis, Missouri; and Dallas, Texas. The following tracers were selected: potassium (K) for biomass burning PM_2.5_, elemental carbon (EC) for diesel PM_2.5_, zinc (Zn) for gasoline PM_2.5_, silicon (Si) for dust PM_2.5_, and organic carbon (OC) for both mobile and burning PM_2.5_.

For each city, we estimated associations between tracer constituents and respiratory disease ED visits to assess consistency with the associations observed for source-specific PM_2.5_ (Figure 2). For biomass burning PM_2.5_, the observed patterns of associations across cities and lags were similar to the patterns observed for potassium and OC, which are tracers for PM_2.5_ from biomass burning. Although we did not observe positive associations for diesel vehicles in Atlanta and Birmingham, we found some positive associations between EC and ED visits in these cities. EC, though generally a better tracer for diesel PM_2.5_ than for biomass burning PM_2.5_, was moderately correlated with biomass burning PM_2.5_ in these cities (0.42 and 0.47, respectively). There was little evidence of association for zinc, a tracer for gasoline PM_2.5_, or silicon, a tracer for dust PM_2.5_, consistent with the source-specific results.

### Sensitivity Analysis

We found that estimated health effect associations for subcategories of respiratory diseases had wider confidence intervals than those for combined respiratory diseases because there were fewer daily counts for each subcategory (see Figures S3–S6). We found some evidence of associations between PM_2.5_ from biomass burning and URI in all cities except Dallas, although the lag corresponding to the largest associations varied between cities.

We found that results were mostly consistent across models with varying confounder control, although our estimated relative risks were frequently greater in magnitude in models without control for weekdays and holidays (results not shown). We did not find that restricting our analysis to patients residing in the counties closest to each city center and containing the PM_2.5_ monitoring sites substantially changed our results (results not shown). We also did not find that our estimated health effect associations substantially changed when we used a standard CMB approach rather than the EBSP approach for estimating source-specific PM_2.5_.

## Discussion

In a multicity U.S. study that examined the associations between primary source-specific PM_2.5_ and respiratory disease ED visits, we found some evidence of positive associations across cities for PM_2.5_ from biomass burning. The inconsistency in estimated associations for diesel and gasoline vehicles across cities might be driven by the spatial heterogeneity of mobile PM_2.5_ and the placement of monitors relative to roadways in each city. In addition, the large standard errors for PM_2.5_ from diesel and gasoline vehicles in Dallas are likely driven by the relatively low temporal variation in these sources (Table 2). Associations with PM_2.5_ from dust were smaller in magnitude and were frequently consistent with the null across cities. The lags where the associations were largest in magnitude varied between cities, which might be driven by between-city differences in hospital use. Between-city differences in estimated health effect associations of source-specific PM_2.5_ could also be driven by differences in their respective populations, including air conditioning use and susceptibility (Bell et al. 2009; Ostro et al. 2008), or by differential exposure error.

Previous studies have estimated associations between respiratory morbidity and source-specific PM_2.5_. Sarnat et al. (2008) did not find evidence of positive associations between respiratory disease ED visits and PM_2.5_ from gasoline vehicles, diesel vehicles, wood smoke, or soil in Atlanta, but they used same-day exposure and had a shorter time frame than was available in the present study. Andersen et al. (2007) found that PM < 10 μm (PM_10_) from biomass burning was associated with increased respiratory hospital admissions in Copenhagen, Denmark. Gass et al. (2015) found positive associations between pediatric asthma ED visits in Atlanta and gasoline and diesel PM_2.5_; these associations were larger in magnitude than those found for biomass burning PM_2.5_. Other studies have found evidence of associations between respiratory hospitalizations and traffic PM_2.5_ (Ito et al. 2013) and road dust PM_2.5_ (Bell et al. 2014), although these studies did not identify biomass burning as a source of PM_2.5_.

We observed positive associations between biomass burning PM_2.5_ and respiratory ED visits, which corresponded well to observed associations for OC and potassium. Although OC is emitted by biomass burning PM_2.5_, OC is also associated with mobile PM_2.5_ including PM_2.5_ from gasoline and diesel vehicles and secondary formation from gaseous emissions. OC consists of many organic compounds that could be used to differentiate the sources of OC, such as levoglucosan as an indicator of biomass burning; however, we did not have daily speciated OC data available for the entirety of this study. Speciated OC data were used in developing the source profiles used in our source apportionment approach (Balachandran et al. 2013), and other studies have used speciated OC data (Zheng et al. 2007). In general, estimated associations for source-specific PM_2.5_ had more uncertainty than estimated associations for PM_2.5_ constituents, likely because source-specific PM_2.5_ is estimated and is not directly measured.

We found that EBSPs for PM_2.5_ from biomass burning, diesel vehicles, gasoline vehicles, and dust were similar across cities, whereas greater differences existed for EBSPs for PM_2.5_ from coal combustion and metals (Table 1; see also Figures S1 and S2). A previous study of the same urban ambient monitors in Atlanta and Birmingham also found the same PM_2.5_ sources to have similar chemical compositions between monitors (Lee et al. 2008). Correlations and nRMSDs are simple tools that can be applied to compare source profiles across cities; however, future work could develop statistical models that provide a more rigorous framework for comparing estimated PM_2.5_ sources across cities.

Although source apportionment models have been primarily developed for data from a single ambient monitor, two previous studies developed source apportionment models for multiple ambient monitors (Jun and Park 2013; Thurston et al. 2011). These models may not be appropriate for multicity epidemiologic studies because they fix source profiles across monitors. For example, in our study, we found that source profiles (EBSPs) for PM_2.5_ from coal combustion and metals varied across cities.

In source apportionment studies, we commonly estimate source-specific PM_2.5_ but do not directly model the known PM_2.5_ sources in each city (e.g., factories). Therefore, some sources estimated using source apportionment might not exactly correspond to existing PM_2.5_ sources. Other methods, such as dispersion modeling, can be used to estimate source-specific PM_2.5_ across a community. However, these methods are generally not applied to time-series data and require information that may not be available for all communities. In contrast, source apportionment models can be readily applied to time series of PM_2.5_ constituent concentrations, which are measured in most urban areas at ambient monitors. Source apportionment studies can also be used to identify groups of PM_2.5_ chemical constituents that are most harmful to human health to help focus future epidemiologic studies on relevant PM_2.5_ sources.

In this analysis, we did not propagate uncertainty from estimating source-specific PM_2.5_ into our estimated health associations. The EBSP approach provides uncertainties associated with estimating source-specific PM_2.5_, and future work could determine how to best incorporate these uncertainties in health effects regression models. Bayesian ensemble-based source apportionment (Balachandran et al. 2013; Gass et al. 2015) and fully Bayesian models (Nikolov et al. 2007) could also be used to propagate the uncertainty from estimating source-specific PM_2.5_.

The approach we developed to compare the chemical composition of source-specific PM_2.5_ across cities can be applied to examine city-to-city heterogeneity in source-specific PM_2.5_ and how it might explain city-to-city heterogeneity in health effects of PM_2.5_ mass. In our study, chi-squared tests of heterogeneity did not reveal that estimated associations for source-specific PM_2.5_ varied across cities; however, longer time series may be needed to fully examine between-city differences. We were unable to examine city-to-city heterogeneity in estimated associations across cities using multilevel models because we were limited to data from four U.S. cities. Although national-level data on ED visits and source-specific PM_2.5_ are not readily available, future work incorporating such data from selected additional cities will be relevant to addressing this objective.

Our study of source-specific PM_2.5_ across four U.S. cities was limited by the amount of available data. We had data from one ambient monitor in each city, which did not allow us to examine spatiotemporal heterogeneity in PM_2.5_ mass or PM_2.5_ constituents across each city. In addition, we only had concentrations of PM_2.5_ chemical constituents to estimate source-specific PM_2.5_ every third day in Birmingham, St. Louis, and Dallas, which limited our ability to fit distributed lag models or models using multiday exposures. Lall et al. (2011) found stronger associations for cardiorespiratory hospital admissions using multiday lagged exposures; therefore, our estimated associations for single-day exposures may be smaller in magnitude than those associated with multiday exposures.

PM_2.5_ constituents have only been collected nationally since 2000 (U.S. EPA 2009), and future work may be able to utilize longer time series to resolve observed differences in estimated associations between cities. Dallas had a shorter time series of data than the other cities investigated herein, with only 332 days of source-specific PM_2.5_ spanning 2006–2009, which led to broad confidence intervals for the estimated associations. For Atlanta and Birmingham, where longer time series were available, we observed somewhat more consistent results across lags (Figures 1 and 2). Longer time series in each city would also improve our ability to estimate associations between source-specific PM_2.5_ and ED visits by age group.

To our knowledge, this is the first multicity study of primary source–specific PM_2.5_ and ED visits. Larger, national-level studies are necessary to inform future NAAQS; however, we have provided a framework for comparing estimated source-specific PM_2.5_ between cities.

## Conclusions

In this multicity study of the associations between primary source–specific PM_2.5_ and ED visits for respiratory diseases, we found some evidence of positive associations across all cities with PM_2.5_ from biomass burning. Associations with PM_2.5_ from diesel and gasoline vehicle sources were less consistent across cities and lags, which could be driven by the spatial heterogeneity of the sources. There was little evidence of association with PM_2.5_ from dust. We found that PM_2.5_ from coal combustion and metal sources varied in chemical composition across cities, which presents challenges for comparing estimated health effect associations between cities. Our approach provides an analytic framework for multicity studies of PM_2.5_ sources to determine those sources most associated with adverse health outcomes and to help inform targeted reduction of ambient PM_2.5_.

## Supplemental Material

(294 KB) PDF
