# Supplementary material for: Associations between Source-Specific Fine Particulate Matter and Emergency Department Visits for Respiratory Disease in Four U.S. Cities
Source: Environ Health Perspect. 2016 Jun 17;125(1):97–103. doi: 10.1289/EHP271 (PMC5226704; doi:10.1289/EHP271)
Supplement: (294 KB) PDF [file EHP271.s001.acco.pdf]

Environ Health Perspect
DOI: 10.1289/EHP271

Note to readers with disabilities: *EHP* strives to ensure that all journal content is accessible to all readers. However, some figures and Supplemental Material published in *EHP* articles may not conform to 508 standards due to the complexity of the information being presented. If you need assistance accessing journal content, please contact ehp508@niehs.nih.gov. Our staff will work with you to assess and meet your accessibility needs within 3 working days.

Supplemental Material

Associations between Source-Specific Fine Particulate Matter and Emergency Department Visits for Respiratory Disease in Four U.S. Cities

Jenna R. Krall, James A. Mulholland, Armistead G. Russell, Sivaraman Balachandran, Andrea Winquist, Paige E. Tolbert, Lance A. Waller, and Stefanie Ebelt Sarnat

Table of Contents

Figure S1 Ensemble-based source profiles (EBSPs) for summer source-specific $PM_{2.5}$ corresponding to sources of primary $PM_{2.5}$ for Atlanta, GA; Birmingham, AL; St. Louis, MO; and Dallas, TX. The EBSPs are unitless but can be roughly interpreted as the amount (in $\mu g/m^3$) of each chemical species per $\mu g/m^3$ of source-specific $PM_{2.5}$.

Figure S2 Ensemble-based source profiles (EBSPs) for winter source-specific $PM_{2.5}$ corresponding to sources of primary $PM_{2.5}$ for Atlanta, GA; Birmingham, AL; St. Louis, MO; and Dallas, TX. The EBSPs are unitless but can be roughly interpreted as the amount (in $\mu g/m^3$) of each chemical species per $\mu g/m^3$ of source-specific $PM_{2.5}$.

Table S1 Mean (minimum, maximum) correlation between daily concentrations of $PM_{2.5}$ mass and source-specific $PM_{2.5}$ across Atlanta, GA; Birmingham, AL; St. Louis, MO; and Dallas, TX.

Table S2 Mean (standard deviation) number of daily emergency department visits for respiratory diseases and subcategories of respiratory disease for Atlanta, GA; Birmingham, AL; St. Louis, MO; and Dallas, TX.

Table S3 Number of days of available data for selected tracer $PM_{2.5}$ chemical constituents for Atlanta, GA; Birmingham, AL; St. Louis, MO; and Dallas, TX. Tracers were selected as potassium (K) for biomass burning $PM_{2.5}$, EC for diesel $PM_{2.5}$, zinc (Zn) for gasoline $PM_{2.5}$, silicon (Si) for dust $PM_{2.5}$, as well as OC for both mobile and burning $PM_{2.5}$.

Table S4 Average (standard deviation) concentration and median of city-specific interquartile ranges (IQR) in $\mu g/m^3$ for selected tracer $PM_{2.5}$ chemical constituents in Atlanta, GA; Birmingham, AL; St. Louis, MO; and Dallas, TX. Tracers were selected as potassium (K) for biomass burning $PM_{2.5}$, EC for diesel $PM_{2.5}$, zinc (Zn) for gasoline $PM_{2.5}$, silicon (Si) for dust $PM_{2.5}$, as well as OC for both mobile and burning $PM_{2.5}$.

Table S5 Mean (minimum, maximum) correlation between daily concentrations of selected tracer $PM_{2.5}$ chemical constituents across for Atlanta, GA; Birmingham, AL; St. Louis, MO; and Dallas, TX. Tracers were selected as potassium (K) for biomass burning $PM_{2.5}$, EC for diesel $PM_{2.5}$, zinc (Zn) for gasoline $PM_{2.5}$, silicon (Si) for dust $PM_{2.5}$, as well as OC for both mobile and burning $PM_{2.5}$.

Table S6 Mean (minimum, maximum) correlation between daily concentrations of $PM_{2.5}$ mass, source-specific $PM_{2.5}$, and selected tracer $PM_{2.5}$ chemical constituents across Atlanta, GA; Birmingham, AL; St. Louis, MO; and Dallas, TX. Tracers were selected as potassium (K) for biomass burning $PM_{2.5}$, EC for diesel $PM_{2.5}$, zinc (Zn) for gasoline $PM_{2.5}$, silicon (Si) for dust $PM_{2.5}$, as well as OC for both mobile and burning $PM_{2.5}$.

Figure S3 Estimated relative risks of pneumonia ED visits for interquartile range increases (IQR) in $PM_{2.5}$ mass and source-specific $PM_{2.5}$ using single day exposure lags 0 to 3 for Atlanta, GA; Birmingham, AL; St. Louis, MO; and Dallas, TX.

Figure S4 Estimated relative risks of chronic obstructive pulmonary disease ED visits for interquartile range increases (IQR) in $PM_{2.5}$ mass and source-specific $PM_{2.5}$ using single day exposure lags 0 to 3 for Atlanta, GA; Birmingham, AL; St. Louis, MO; and Dallas, TX.

Figure S5 Estimated relative risks of upper respiratory infection ED visits for interquartile range increases (IQR) in $PM_{2.5}$ mass and source-specific $PM_{2.5}$ using single day exposure lags 0 to 3 for Atlanta, GA; Birmingham, AL; St. Louis, MO; and Dallas, TX.

Figure S6 Estimated relative risks of asthma/wheeze ED visits for interquartile range increases (IQR) in $PM_{2.5}$ mass and source-specific $PM_{2.5}$ using single day exposure lags 0 to 3 for Atlanta, GA; Birmingham, AL; St. Louis, MO; and Dallas, TX.

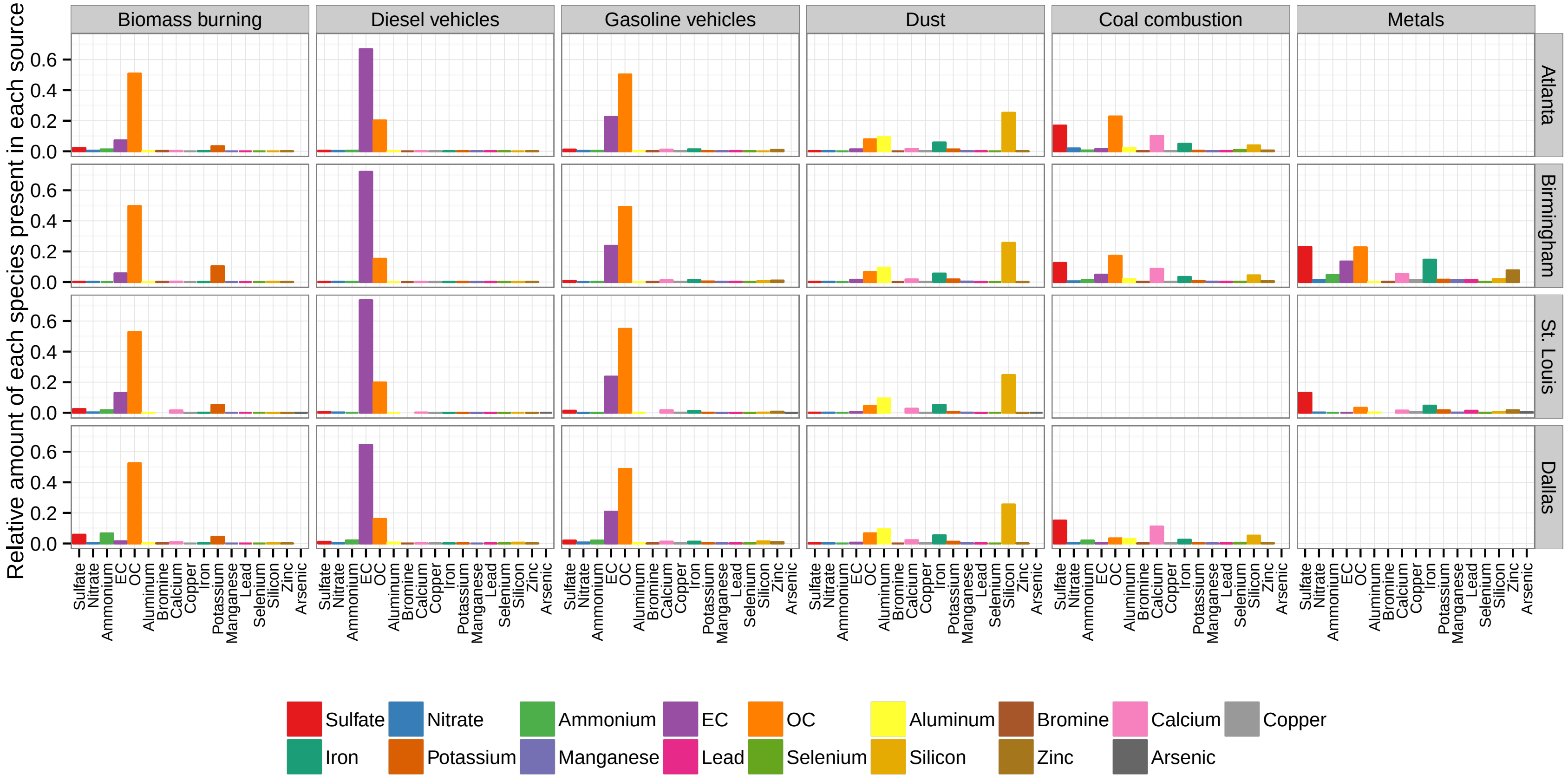

Figure S1: Ensemble-based source profiles (EBSPs) for summer source-specific $PM_{2.5}$ corresponding to sources of primary $PM_{2.5}$ for Atlanta, GA; Birmingham, AL; St. Louis, MO; and Dallas, TX. The EBSPs are unitless but can be roughly interpreted as the amount (in $\mu g/m^3$) of each chemical species per $\mu g/m^3$ of source-specific $PM_{2.5}$.

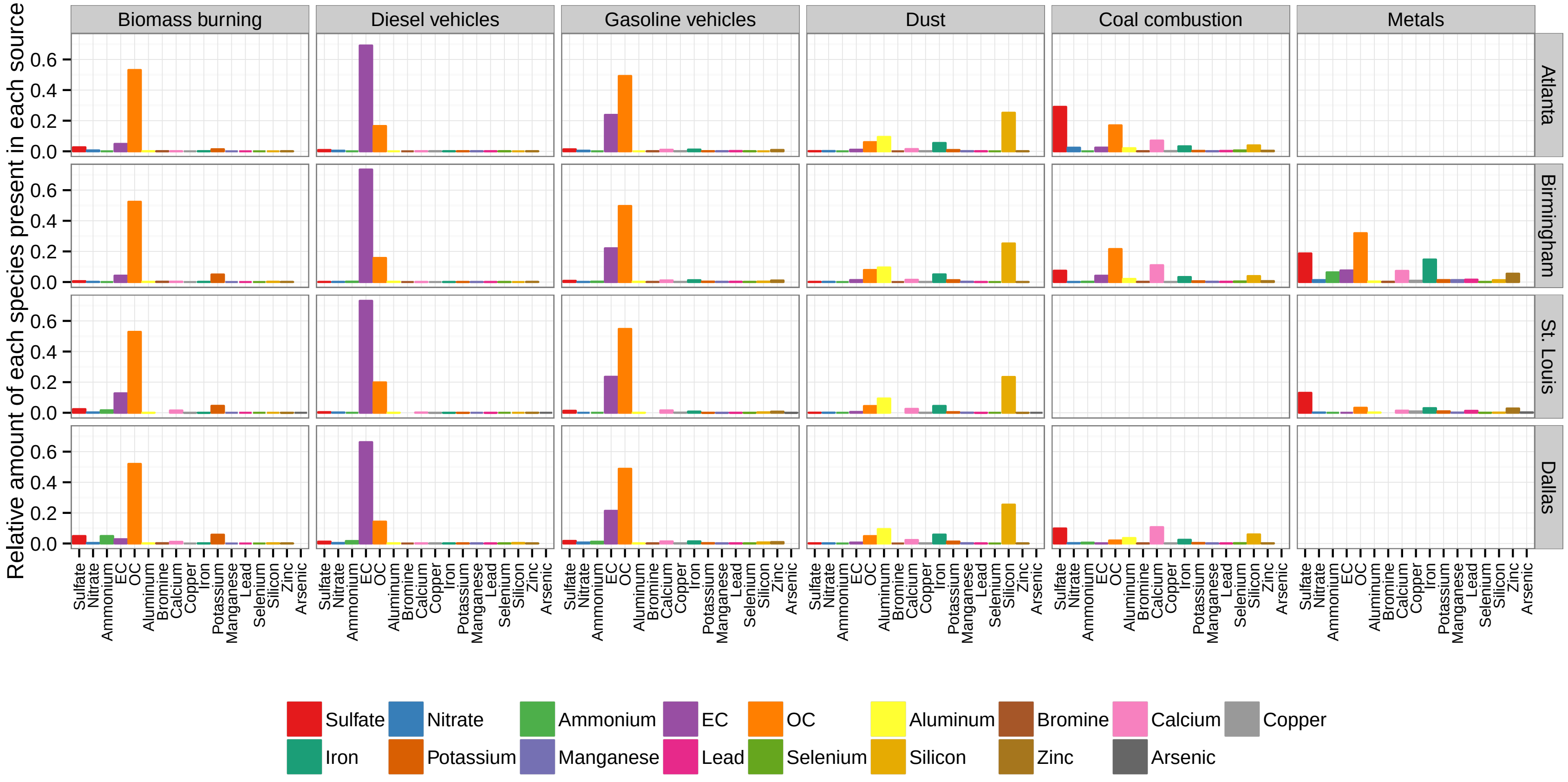

Figure S2: Ensemble-based source profiles (EBSPs) for winter source-specific $PM_{2.5}$ corresponding to sources of primary $PM_{2.5}$ for Atlanta, GA; Birmingham, AL; St. Louis, MO; and Dallas, TX. The EBSPs are unitless but can be roughly interpreted as the amount (in $\mu g/m^3$) of each chemical species per $\mu g/m^3$ of source-specific $PM_{2.5}$.

Table S1: Mean (minimum, maximum) correlation between daily concentrations of $PM_{2.5}$ mass and source-specific $PM_{2.5}$ across Atlanta, GA; Birmingham, AL; St. Louis, MO; and Dallas, TX.

	Biomass burning	Diesel vehicles	Gasoline vehicles	Dust	Coal combustion[a]	Metals[b]
$PM_{2.5}$ mass	0.38 (0.24, 0.55)	0.30 (0.18, 0.43)	0.26 (0.12, 0.37)	0.24 (0.12, 0.34)	0.29 (0.06, 0.55)	0.28 (0.11, 0.44)
Biomass burning		0.17 (0.08, 0.36)	0.23 (0.00, 0.57)	0.09 (0.02, 0.20)	0.09 (-0.01, 0.19)	0.23 (0.05, 0.41)
Diesel vehicles			0.36 (0.26, 0.53)	0.04 (0.01, 0.09)	0.18 (0.09, 0.25)	0.26 (0.21, 0.30)
Gasoline vehicles				0.01 (-0.13, 0.19)	0.28 (0.24, 0.35)	0.26 (0.05, 0.47)
Dust					0.10 (-0.03, 0.28)	0.10 (0.02, 0.18)
Coal combustion						0.23[c] (– , –)

[a]Correlations for three cities: Atlanta, Birmingham, and Dallas
[b]Correlations for two cities: Birmingham and St. Louis
[c]Only Birmingham identified both $PM_{2.5}$ from coal combustion and metals

Table S2: Mean (standard deviation) number of daily emergency department visits for respiratory diseases and subcategories of respiratory disease for Atlanta, GA; Birmingham, AL; St. Louis, MO; and Dallas, TX.

	Atlanta	Birmingham	St. Louis	Dallas
Respiratory	361 (129)	59 (27)	281 (81)	455 (159)
Pneumonia	47 (20)	8 (4)	45 (16)	72 (27)
COPD	18 (7)	6 (3)	16 (5)	23 (7)
Upper Respiratory Infection	212 (78)	36 (18)	162 (52)	263 (100)
Asthma/Wheeze	72 (29)	9 (5)	51 (15)	77 (24)

Table S3: Number of days of available data for selected tracer $PM_{2.5}$ chemical constituents for Atlanta, GA; Birmingham, AL; St. Louis, MO; and Dallas, TX. Tracers were selected as potassium (K) for biomass burning $PM_{2.5}$, EC for diesel $PM_{2.5}$, zinc (Zn) for gasoline $PM_{2.5}$, silicon (Si) for dust $PM_{2.5}$, as well as OC for both mobile and burning $PM_{2.5}$.

	Atlanta	Birmingham	St. Louis	Dallas
K	3206	733	722	330
OC	3618	808	728	332
EC	3621	808	728	332
Zn	3206	733	722	330
Si	3206	733	722	330

Table S4: Average (standard deviation) concentration and median of city-specific interquartile ranges (IQR) in μg/m^3 for selected tracer $PM_{2.5}$ chemical constituents in Atlanta, GA; Birmingham, AL; St. Louis, MO; and Dallas, TX. Tracers were selected as potassium (K) for biomass burning $PM_{2.5}$, EC for diesel $PM_{2.5}$, zinc (Zn) for gasoline $PM_{2.5}$, silicon (Si) for dust $PM_{2.5}$, as well as OC for both mobile and burning $PM_{2.5}$.

	Atlanta	Birmingham	St. Louis	Dallas	IQR
K	0.03 (0.02)	0.05 (0.04)	0.07 (0.05)	0.03 (0.02)	0.03
OC	3.93 (2.20)	5.93 (3.19)	4.46 (1.87)	2.82 (1.15)	2.34
EC	1.37 (0.96)	1.16 (0.83)	0.83 (0.45)	0.55 (0.31)	0.61
Zn	0.01 (0.01)	0.09 (0.14)	0.03 (0.03)	0.01 (0.00)	0.02
Si	0.09 (0.10)	0.21 (0.24)	0.11 (0.18)	0.16 (0.28)	0.08

Table S5: Mean (minimum, maximum) correlation between daily concentrations of selected tracer $PM_{2.5}$ chemical constituents across for Atlanta, GA; Birmingham, AL; St. Louis, MO; and Dallas, TX. Tracers were selected as potassium (K) for biomass burning $PM_{2.5}$, EC for diesel $PM_{2.5}$, zinc (Zn) for gasoline $PM_{2.5}$, silicon (Si) for dust $PM_{2.5}$, as well as OC for both mobile and burning $PM_{2.5}$.

	OC	EC	Zn	Si
K	0.50 (0.30, 0.58)	0.34 (0.08, 0.50)	0.19 (0.07, 0.30)	0.54 (0.38, 0.66)
OC		0.64 (0.44, 0.80)	0.30 (0.14, 0.40)	0.18 (0.06, 0.28)
EC			0.46 (0.20, 0.65)	0.12 (-0.05, 0.29)
Zn				0.09 (-0.11, 0.40)

Table S6: Mean (minimum, maximum) correlation between daily concentrations of $PM_{2.5}$ mass, source-specific $PM_{2.5}$, and selected tracer $PM_{2.5}$ chemical constituents across Atlanta, GA; Birmingham, AL; St. Louis, MO; and Dallas, TX. Tracers were selected as potassium (K) for biomass burning $PM_{2.5}$, EC for diesel $PM_{2.5}$, zinc (Zn) for gasoline $PM_{2.5}$, silicon (Si) for dust $PM_{2.5}$, as well as OC for both mobile and burning $PM_{2.5}$.

	K	OC	EC	Zn	Si
$PM_{2.5}$ mass	0.50 (0.39, 0.63)	0.66 (0.51, 0.81)	0.47 (0.20, 0.64)	0.28 (0.25, 0.30)	0.27 (0.14, 0.37)
Biomass burning	0.74 (0.60, 0.84)	0.44 (0.32, 0.52)	0.32 (0.14, 0.47)	0.19 (0.10, 0.41)	0.16 (0.04, 0.36)
Diesel vehicles	0.20 (0.15, 0.26)	0.47 (0.35, 0.62)	0.71 (0.67, 0.81)	0.29 (0.10, 0.41)	0.06 (0.02, 0.12)
Gasoline vehicles	0.24 (0.03, 0.44)	0.42 (0.34, 0.55)	0.53 (0.45, 0.64)	0.48 (0.29, 0.64)	0.05 (-0.12, 0.29)
Dust	0.46 (0.38, 0.55)	0.16 (0.10, 0.23)	0.10 (-0.05, 0.23)	0.01 (-0.11, 0.16)	0.91 (0.74, 0.98)
Coal combustion[a]	0.18 (0.06, 0.37)	0.30 (0.21, 0.47)	0.30 (0.13, 0.49)	0.18 (0.15, 0.23)	0.16 (0.06, 0.34)
Metals[b]	0.29 (0.14, 0.43)	0.34 (0.25, 0.44)	0.30 (0.12, 0.47)	0.38 (0.08, 0.67)	0.20 (0.04, 0.37)

[a]Correlations for three cities: Atlanta, Birmingham, and Dallas
[b]Correlations for two cities: Birmingham and St. Louis

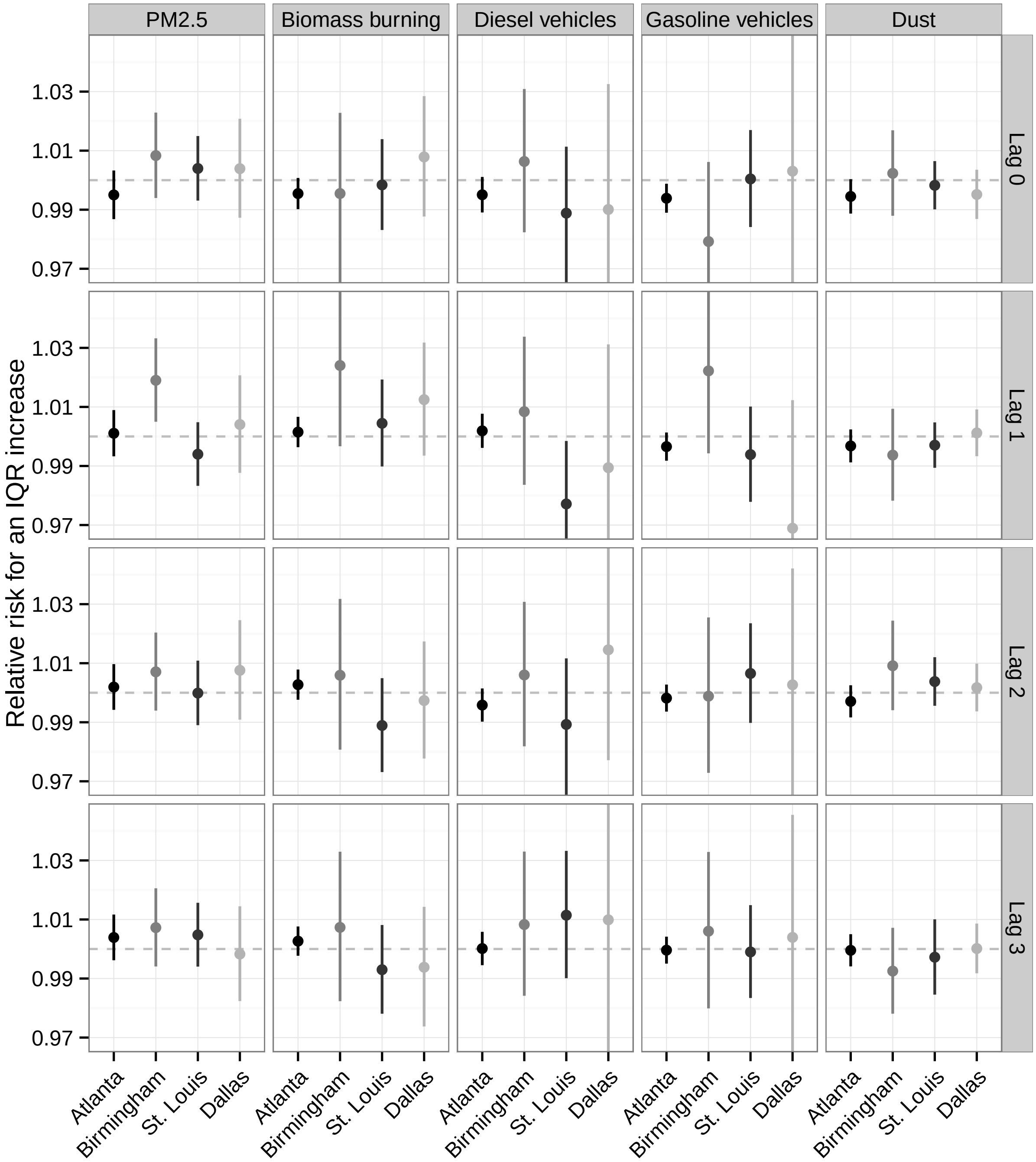

Figure S3: Estimated relative risks of pneumonia ED visits for interquartile range increases (IQR) in $PM_{2.5}$ mass and source-specific $PM_{2.5}$ using single day exposure lags 0 to 3 for Atlanta, GA; Birmingham, AL; St. Louis, MO; and Dallas, TX.

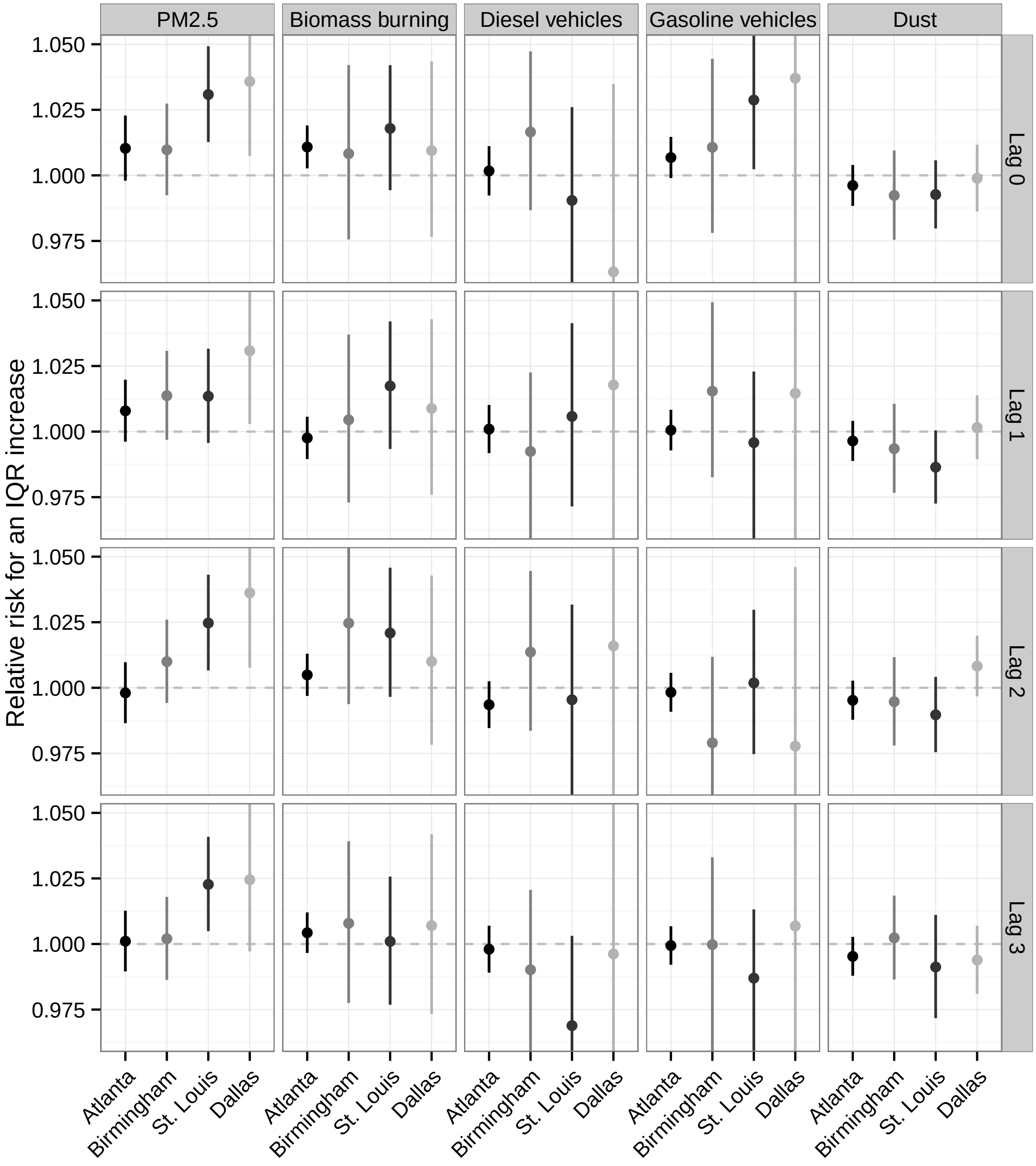

Figure S4: Estimated relative risks of chronic obstructive pulmonary disease ED visits for interquartile range increases (IQR) in $PM_{2.5}$ mass and source-specific $PM_{2.5}$ using single day exposure lags 0 to 3 for Atlanta, GA; Birmingham, AL; St. Louis, MO; and Dallas, TX.

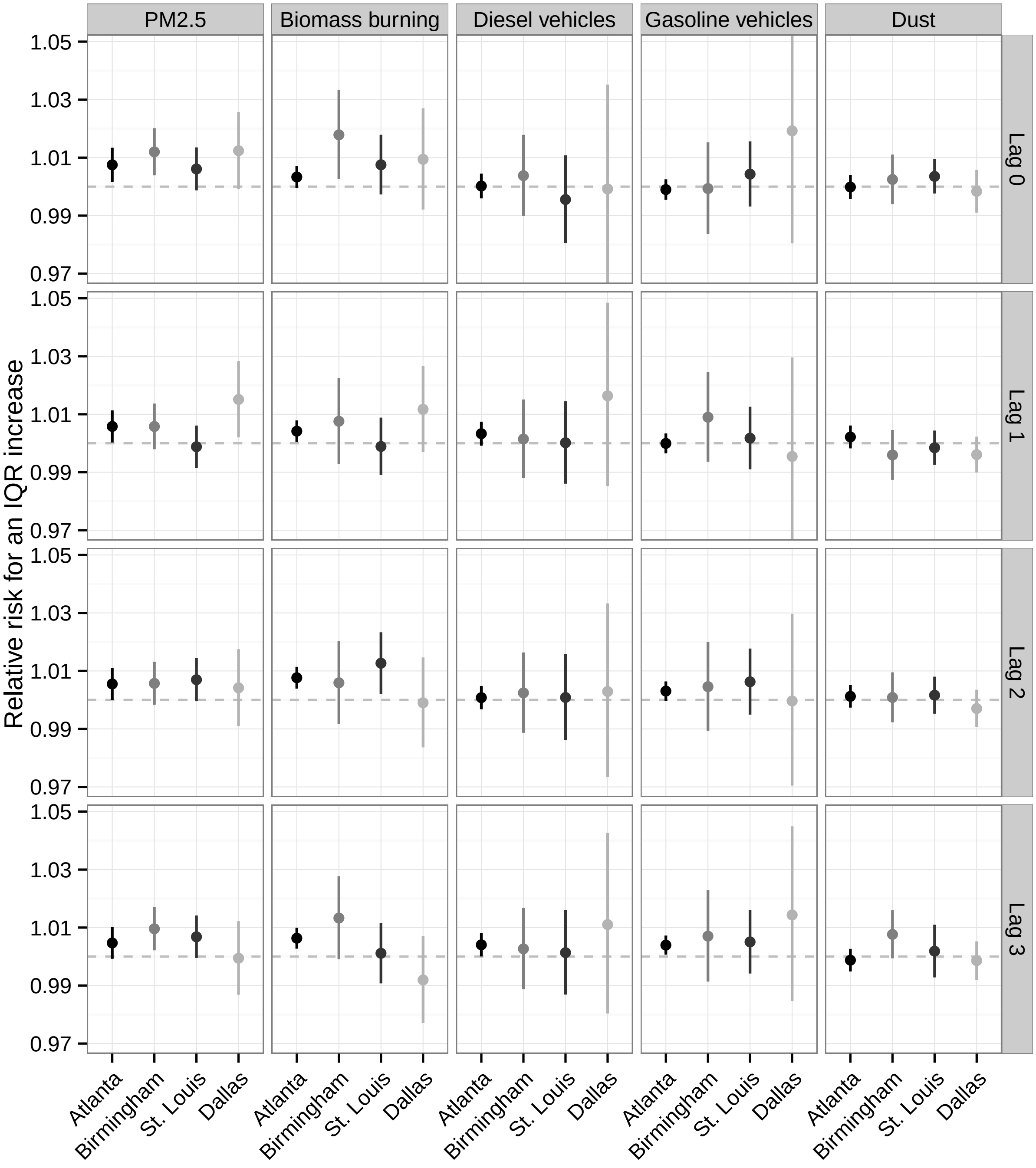

Figure S5: Estimated relative risks of upper respiratory infection ED visits for interquartile range increases (IQR) in $PM_{2.5}$ mass and source-specific $PM_{2.5}$ using single day exposure lags 0 to 3 for Atlanta, GA; Birmingham, AL; St. Louis, MO; and Dallas, TX.

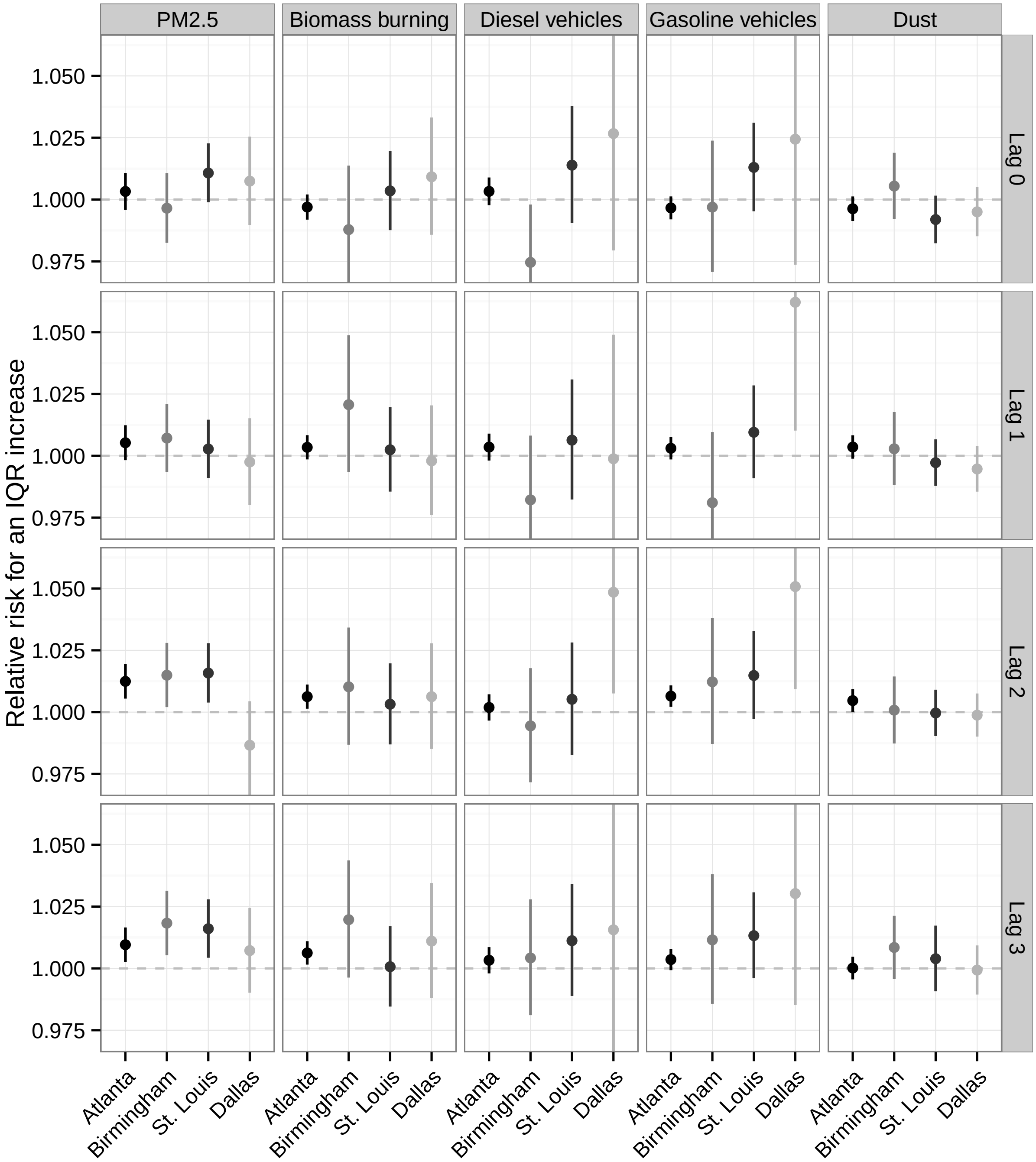

Figure S6: Estimated relative risks of asthma/wheeze ED visits for interquartile range increases (IQR) in $PM_{2.5}$ mass and source-specific $PM_{2.5}$ using single day exposure lags 0 to 3 for Atlanta, GA; Birmingham, AL; St. Louis, MO; and Dallas, TX.
